# Factors Associated with Occupational Needle Stick and Sharps Injuries among Hospital Healthcare Workers in Bale Zone, Southeast Ethiopia

**DOI:** 10.1371/journal.pone.0140382

**Published:** 2015-10-15

**Authors:** Tolesa Bekele, Alem Gebremariam, Muhammedawel Kaso, Kemal Ahmed

**Affiliations:** 1 Department of Public Health, College of Medicine and Health Sciences, Madda Walabu University, Bale-Goba, Oromia, Ethiopia; 2 Department of Public Health, College of Medicine and Health Sciences, Adigrat University, Adigrat, Tigray, Ethiopia; Alberta Provincial Laboratory for Public Health/ University of Alberta, CANADA

## Abstract

**Background:**

Needle stick and sharps injuries are occupational hazards to healthcare workers. Every day healthcare workers are exposed to deadly blood borne pathogens through contaminated needles and other sharp objects. About twenty blood borne pathogens can be transmitted through accidental needle stick and sharp injury. The study was conducted to determine the lifetime and past one year prevalence of needle stick and sharps injuries and factors associated with the past one year injuries among hospital healthcare workers in Southeast Ethiopia.

**Methods:**

An institutional based cross sectional study was conducted in December 2014 among healthcare workers in four hospitals of Bale zone, Southeast of Ethiopia. A total of 362 healthcare workers were selected randomly from each department in the hospitals. Data were collected using self-administered questionnaire. The collected data were entered into Epi-Info version 3.5 and analyzed using SPSS version 20.0. Multivariable logistic regression analysis was used to identify the independent effect of each independent variable on the outcome variable. Written informed consent was secured from the participants.

**Results:**

The prevalence of lifetime needle stick and sharp injury was 37.1% with 95% CI of 32.0% to 42.5%. The prevalence of injury within the past one year was 19.1% with 95% CI of 14.9% to 23.3%. Emergency ward was a department with highest needle stick and sharp injury (31.7%). The main cause of injury was syringe needles (69.8%). Participants who practiced needle recapping had higher odds of needle stick and sharp injury within the past 12 months (AOR = 3.23, 95% CI: 1.78, 5.84) compared to their counterparts.

**Conclusions:**

Nearly one out of five respondents had experienced needle stick and/or sharp injury at least once within past one year. There were practices and behaviors that put healthcare workers at risk of needle stick and sharp injury at the study area. Needle recapping was key modifiable risk behavior. Health policy makers and hospital administrators should formulate strategies to improve the working condition for healthcare workers and increase their adherence to universal precautions.

## Introduction

Sharp injury occurs when sharp instruments such as needle penetrates the skin. If the sharp instrument is contaminated with blood and body fluids, there is potential for transmission of infection. Globally, more than 35 million healthcare workers (HCWs) are suffering from occupational needle sick and sharp injury (NSSI) every year [1]. While as many as twenty blood borne pathogens (BBPs) can be transmitted by accidental injury, the potential life threatening are Human Immunodeficiency Virus (HIV), hepatitis B virus (HBV) and hepatitis C virus (HCV) [2]. Moreover, HBV is highly contagious and infects one out of three people [3].

A series of precautions were proposed for preventing occupational exposures and for handling of potentially infectious materials of blood and body fluids. These series of effective precautions designed to protect healthcare workers from infection with a range of pathogens of blood and body fluids are known as universal precautions (UPs). This is universally recognized that healthcare workers (HCWs) should practice no two handed recapping of needles, safe collection and disposal of needles and sharps with required puncture and liquid proof safety boxes in each patient care areas, wearing gloves for contact with blood and body fluids, non-intact skin and mucous membrane, promptly and carefully cleaning up spills of blood and other body fluids and using safety system for healthcare waste management and disposal [4].

Worldwide, the number of HCWs annually exposed to sharps injuries contaminated with HBV, HCV or HIV, was estimated to be 2.1 million, 926, 000, and 327, 000, respectively [4]. In developing countries, 40–60% of HBV infection among HCWs was attributed to professional hazard while in developed countries the attributed fraction was less than 10% due to vaccination coverage [5]. Even though up to 90% of these injuries occur in developing nations, the number of studies reporting this serious issue is less compared to developed nations [6]. In less developed countries, the risk of occupational transmission due to BBPs is increased due to excessive handling of contaminated needles that result from some common unsafe practices. These include administration of unnecessary injections on demand, reuse of non-sterile needles when supplies are low and inappropriate disposal of hazardous waste [7].

Some HCWs are at greater risk of acquiring infections through sharps injuries than others. These include those who are in close contact with body fluids such as surgeons, obstetricians, midwives and laboratory personnel [8]. Despite of the long-standing recommendations for high risk group vaccination against HBV, it remains unavailable to HCWs in most resource-limited settings in Sub-Saharan Africa and even when available, the coverage remains low [9].

Neither the prevalence of NSSI nor the factors associated with it have been well understood among HCWs in Sub-Saharan Africa [10]. Literatures revealed that there are many factors that contribute to NSSI among HCWs. Such factors are irregular utilization of protective gear, type of occupation of healthcare workers, disposing of used needle, injecting medicine, recapping of needles and drawing of blood [11–12]. Moreover, healthcare workers who followed universal precautions were 66% less likely to have NSSIs than those who did not adhere to these recommendations [13].

In Ethiopia, concrete knowledge about transmission of infection in healthcare facilities due to NSSI is limited. It varies from setting to setting and unsafe practices are common. Little is known about the prevalence of NSSI in Ethiopian healthcare facilities. A few studies reported high prevalence of NSSI in central and northern part of the country. However, contributing factors to the occurrences of occupational NSSI among HCWs was not yet well addressed. Therefore, it is an important to determine and document the lifetime and past one year prevalence of needle stick and sharps injuries, and associated factors with the past one year injuries among hospital healthcare workers in Southeast Ethiopia.

## Methods

### Study area and design

The study was carried out in December 2014 in four hospitals namely Ginir, Robe, Delo mena and Goba in Bale Zone, Southeast of Ethiopia. By the year 2014, the four hospitals were offering different types of healthcare services for the surrounding community. Facility based cross sectional study design was employed. All individuals working in four hospitals who have direct contact with patients and/or equipments used on patients were involved in the study. These workers were doctors, anesthetists, health officers, nurses, midwives, laboratory personnel, laundry workers and waste handlers. Individuals who were on annual leave during data collection time and those who could not respond to the questions due to illness were not included in the study.

### Sample size and sampling procedure

The sample size was determined using single population proportion formula. It was computed by considering 31.1% of past 12 months prevalence of needle stick and sharp injury [14]; 95% confidence level, and 0.05 margin of error. This was found 329 and adding 10% allowance for non-response rate, the total sample size was 362. All hospitals in Bale zone were included in the study. Before selection of study participants first we obtained the list of the workers and categorized them into their specific working department. Then proportionate allocation to size method was used for each department in the hospitals to share the total sample size. Study participants who fulfilled the inclusion criteria were selected by simple random sampling technique using the list of the workers from each working department in the hospitals.

### Data collection procedure

Questionnaire was adopted and developed with modification from related studies [14–16]. First, questionnaire was prepared in English then it was translated to Afan Oromo and Amharic (local languages) by experts in both languages and then retranslated to English to check for consistency. Eight data collection facilitators (BSc nurses) were assigned to the four hospitals (two per hospital). They have been trained for one day on the study instrument and data collection procedures. Questionnaire was pretested on 5% of the same source population other than the sampled population in Goba and Robe hospitals. Based on the pre-test; questions were revised, edited and those found to be unclear or confusing were modified. Finally, data were collected using structured self-administered questionnaire. The questionnaire included: (i) background information of the respondents, (ii) circumstances in which the injuries occurred and (iii) participants’ practice of infection prevention (See S1 File). Supervision during data collection was done by investigators to see how data collection facilitators were handling the data collection. Filled questionnaires were checked daily for completeness, legibility and consistency.

### Data analysis

The collected data were checked for completeness and consistency by the investigators ahead of data entry for analysis. Completed questionnaires were given identification numbers and entered into Epi Info version 3.5. Twenty percent of the entered questionnaires were double checked for the purpose of comparing entered data with the actual questionnaire. Data were cleaned for missing values by running frequencies and outliers by computing standard scores. Cleaned data were exported from Epi Info to Statistical Package for Social Sciences (SPSS) version 20.0 for analysis. Descriptive statistics were computed and presented in the form of texts and tables. A binary outcome variable indicating “have you ever experienced or faced needle stick or sharps injuries at your work place within the past one year?” The response was coded as “yes” and “no” and it was used as the dependent variable. Bi-variate analysis was used to determine the association between independent and outcome variables. Multivariable logistic regression model was used to identify the relative importance of each predictor to the dependent variable by controlling for the effect of other variables. Those variables which were potential independent predictors on bi-variate analysis with P-value < 0.3 were entered to multivariable logistic regression analysis. The association between dependent and independent variables was determined using odds ratio (OR) with 95% confidence interval (CI). The level of significance was taken at α = 0.05.

### Ethical statement

Ethical clearance was obtained from the Ethical and Review Committee of Madda Walabu University, College of Medicine and Health Sciences. An official letter of permission was obtained from Madda Walabu University Research and Community Service Directorate Office and given to each hospital. Written consent was secured from each participant. Participants were told that information provided was confidential and that their identities were not revealed in association with the information they provided.

## Results

### Socio-demographic characteristics of participants

A total of 340 hospital healthcare workers responded fully to the self-administered questionnaire providing a response rate of 93.9%. The mean age of the respondents was 28.3 (SD±7.7) years. Fifty five percent of the respondents were females. More than half 187 (55.0%) of the respondents were married at the time of data collection. Majority of the participants were Orthodox Christian by religion (62.1%) and Oromo by ethnicity (79.1%). More than three forth 285 (83.5%) of workers were graduated from college or university with diploma and above. Seven out of ten (72.6%) HCWs had five years and below work experience. The mean time of work experience was 45.90 ± 45.15 SD months ranging from 2 to 360 months. Nearly half (49.7%) of the respondents were nurses by profession Table 1.

**Table 1:** Socio-demographic characteristics of hospital healthcare workers in Bale zone, December, 2014 (n = 340).

Socio- demographic characteristics	Frequency (n)	Percentage (%)
**Number of HCWs participated on study**		
Ginir hospital	90	26.5
Goba hospital	115	33.8
Delo mena hospital	70	20.6
Robe hospital	65	19.1
**Educational level**		
Grade(5–8^th^)	3	0.9
Grade(9–12^th^)	52	15.3
College diploma and above	285	83.8
**Professional category**		
Nurse	169	49.7
Physician	26	7.6
Midwife	33	9.7
Anesthesia	7	2.1
Health officer	8	2.4
Clinical laboratory	22	6.5
Laundry worker	13	3.8
Janitor/Cleaner	62	18.2
**Working department**		
Emergency unit	25	7.4
Pediatric ward	29	8.5
Maternity ward	60	17.6
Operation theatre unit	23	6.8
Medical ward	28	8.2
Surgical ward	21	6.2
Waste handler unit	62	18.2
Laboratory unit	22	6.5
Outpatient department	56	16.5
Laundry room	14	4.1

### Prevalence and circumstances of needle stick and sharp injury

The overall prevalence of NSSI was 37.1% with 95% CI of 32.0% to 42.5%. The rate of injury within past 12 months, 24 months and 3 years and above were 19.1% (95% CI of 14.9% to 23.3%), 9.1% (95% CI of 6.0% to 12.2%), and 8.8% (95% CI of 5.8% to 11.8%), respectively. From the total of respondents who had ever experienced occupational injury, 55 (43.7%) were exposed more than once. Nearly one third (31.7%) of the injuries occurred in emergency unit. Other injuries occurred in medical ward (17.5%), maternity ward (13.5%), surgical ward (9.5%), paediatrics (8.7%), operation theatre unit (8.7%) and others such as laboratory unit, outpatient department, vaccination and TB clinic were responsible for 10.4% of injuries. Regarding materials which caused injury, the highest proportion was by syringe needles (69.8%), followed by suture needle (15.9%). The degree of injury accounted by superficial was 64.3% among injuries.

Majority (59.5%) of the materials caused injury was used on patients and 24.6% were from unknown sources whether it was used on patients or not. Nearly half (52%) of the materials which caused injury were used on patients with known type of cases. These cases were HIV patients (35.9%) followed by HBV patients (17.9%). The rest 43.6% were from non blood borne disease cases. Regarding the practice of HCWs on job, 36.5% of the respondents had recapped needles after use at least once during their work time. More than half (55.6%) of the needles were recapped using one hand. More than one quarter (27.7%) of the HCWs did not follow universal precautions and 2.4% did not wear protective gear during day to day activities in their respective working department. Nearly two third (65.3%) of the healthcare workers had not taken training on infection prevention at the time of interview. Most of injuries were occurred during needle recapping (46%) followed by opening needle cap (21.4%), disposal and cleaning the work area (16.7%) and during washing instrument (14.3%) Table 2.

**Table 2:** Circumstances in which needle stick and sharps injury occurrence among hospital workers in Bale zone, December, 2014 (n = 126).

Reasons for needle or sharp injury	Frequency (n)	Percentage (%)*
During needle recapping	58	46.0
During opening the needle cap	27	21.4
During disposal and cleaning the work area	21	16.7
During washing instrument	18	14.3
Improperly disposed needle	14	11.1
During giving injection to patient	13	10.3
Sudden movement of patient	12	9.5
Lack of concentration	10	7.9
Drawing blood from patient	9	7.1
During collection of clothes for laundry	7	5.6
During blood transferring into test tube	5	4.0
Attempting to bend the needle	3	2.4

* Each of the percentages does not add up to 100% because respondents could choose several responses which could be more than one reasons

### Needle stick and sharp injury within the past 12 months among HCWs

The highest prevalence of occupational NSSI was observed among HCWs who practiced needle recap after use (32.3%) compared to those who do not have history of recap (11.6%). This difference was statistically significant. The occurrence of NSSI was higher in those who had not taken training on infection prevention (23.9%) compared to those who had taken the training on infection prevention (10.2%). The knowledge of the risk is almost universal (98.8%) among the study subjects. There is also statistically significant difference of NSSI among those who had knowledge about risk of NSSI and those who did not know Table 3.

**Table 3:** Cross-tabulation of prevalence of NSSI within the past one year among HCWs in Bale zone hospitals, December, 2014 (n = 340).

Variables	Frequency of injury in past one year		
	No (%)	Yes (%)	P-value
**Working hospital**			
Ginir	73 (81.1)	17 (18.9)	0.85
Goba	91 (79.1)	24 (20.9)	
Delo mena	59 (84.3)	11 (15.7)	
Robe	52 (80.0)	13 (20.0)	
**Sex of respondents**			
Male	123 (80.4)	30 (19.6)	0.94
Female	152 (81.3)	35 (18.7)	
**Age group of participants**			
≤ 24	94 (79.0)	25 (21.0)	0.81
25–30	122 (81.9)	27 (18.1)	
>30	59 (81.9)	13 (18.1)	
**Educational level**			
High school and below	49 (81.7)	11 (18.3)	0.86
College and above	226 (80.7)	54 (19.3)	
**Professional category**			
Nurse	132 (78.1)	37 (21.9)	0.43
Other professionals (Physicians, Health Officers, Midwifes, Anesthesia & Clinical laboratory)	80 (83.3)	16 (16.7)	
Non-medical (Laundry and Janitor/cleaner)	63 (84.0)	12 (19.1)	
**Total service year**			
<5 years	167 (79.1)	44 (20.9)	**0.3**
> = 5 years	108 (83.7)	21 (16.3)	
**Works in shift**			
Yes	41 (83.7)	8 (16.3)	0.6
No	234 (80.4)	57 (19.6)	
**Know about the risk of NSSI**			
Yes	274 (81.5)	62 (18.5)	0.02
No	1 (25.0)	3 (75.0)	
**Perceived NSSI is avoidable**			
Yes	231(81.9)	51 (18.1)	**0.28**
No	44 (75.9)	14 (24.1)	
**Recap needles after use**			
Yes	84 (67.7)	40 (32.3)	**0.000**
No	191 (88.4)	25 (11.6)	
**Safety guidelines available at working department**			
Yes	184 (84.0)	35 (16.0)	**0.05**
No	91 (75.2)	30 (24.8)	
**Availability of reporting protocol for NSSI in the hospital**			
Yes	159 (82.8)	33 (17.2)	**0.30**
No	116 (78.4)	32 (21.6)	
**Regularly apply universal precautions**			
Yes	198 (80.2)	49 (19.8)	0.58
No	77 (82.8)	16 (17.2)	
**Use personal protective equipments**			
Yes	270 (81.3)	62 (18.7)	**0.18**
No	5 (62.5)	3 (37.5)	
**Ever had training on infection prevention**			
Yes	106 (89.8)	12 (10.2)	**0.002**
No	169 (76.1)	53 (23.9)	

### Factors associated with NSSI within past 12 months

On bi-variate analysis respondents who do not knew about the risk of needle stick and sharp injury were at increased risk of experiencing injury compared to their counterparts. But this was not significant after controlling other variables in multivariable analysis. Respondents who had taken training on infection prevention were less likely to experience NSSI but this was also not significant on multivariable analysis Table 4.

**Table 4:** Multivariable analyses of factors associated with needle stick and sharps injury in the past one year among hospital healthcare workers in Bale zone, December, 2014.

Variables	NSSI within past one year	
	AOR (95% CI)	P-value
**Total service year**		
<5 years	Ref.	
> = 5 years	0.67 (0.36, 1.24)	0.20
**Know about the risk of NSSI**		
Yes	Ref.	
No	9.00 (0.75, 107.00)	0.08
**Perceived NSSI is avoidable**		
Yes	Ref.	
No	1.08 (0.51, 2.30)	0.82
**Recap needles after use**		
Yes	3.23 (1.78, 5.84)	**0.000**
No	Ref.	
**Safety guidelines available at working department**		
Yes	Ref.	
No	1.53 (0.84, 2.76)	0.16
**Availability of reporting protocol for NSSI in the hospital**		
Yes	Ref.	
No	1.10 (0.61, 1.98)	0.74
**Use personal protective equipments**		
Yes	Ref.	
No	2.6 (0.55, 12.29)	0.22
**Ever had training on infection prevention**		
Yes	Ref.	
No	1.8 (0.87, 3.69)	0.10

Ref. = Reference

Respondents who practiced needle recapping were 3 times more likely to experience injuries than who did not recap needle (AOR = 3.23, 95% CI: 1.78, 5.84). Availability of safety guideline, taking training on infection prevention, reporting protocol were not statistically significant. Even though it was not statistically significant, the OR suggests that not having training may be associated with an 80% increased odds of injury in the last one year. Similarly, availability of safety guidelines in the working department, use of personal protective equipments, access to safety guidelines and experience seem to be important Table 4.

## Discussion

In this study the prevalence of lifetime occupational NSSI was 37.1% with 95% CI of 32.0% to 42.5%. This is comparable with the findings from Sub-Saharan Africa (32%) [15]. But the prevalence in this study is lower than the figure from an earlier studies in Ethiopia where the lifetime proportion was 66.6% in Addis Ababa [17] and 59.0% in Bahir Dar [14]. It cannot be ascertained from the current study whether the prevalence of NSSIs has decreased or if the difference is attributable to the performance of the current study hospitals. The difference may be related to different time of recall periods. The prevalence of NSSIs in the last twelve months before the study was 19.1%, implying that, HCWs in the study hospitals were at risk of contracting blood borne diseases due to NSSI. The past 12 months prevalence in this study was much lower than the healthcare workers in University of Alexandria hospitals (67.9%) [12].

In this study, staff nurses had higher prevalence of NSSI as compared to other healthcare workers. The Ahmadabad’s nurses were the most commonly injured staff among HCWs, constituting 80% of all reported prevalence [18]. This is probably due to the job description of nurses that put them under increased risk of injury such as medication administration, and other procedures which requires the use of needles and other sharp materials.

Overall, emergency unit is the most risky area across the units in hospitals. The study revealed that syringe needle was a major cause of the injuries (69.8%). It is much higher as compared to the study done in Alexandria hospitals (38.4%) [12]. However, it is consistent with a study done in Addis Ababa hospitals 64.5% [17]. This implies that HCWs who had been injured by NSSI might be due to inappropriate needle handling practices. It might be also due to majority of the procedures done for the patients require syringe needles that may put HCWs under risk of injuries.

Regarding the frequency of injury, 43.7% of the respondents had experienced injuries more than one time. This is a little bit lower as compared to the study done in Northern Ethiopia 53.1% [14]. But whatever the difference of the proportions of NSSI, healthcare workers might practice needle recapping after use which may put them under risk of injury. For instance, the prevalence of needle recapping after use in this study was 36.5% and of these 44.4% was recapping using two hands. The practice of recapping is similar to the studies in Nigeria (35.3%) [19] and Northern Ethiopia (34.7%) [14].

Respondents who practiced needle recapping were 3 times more likely to experience injury than who did not recap needles after use. Recapping needles after use was positively associated with NSSI in previous studies [11, 17]. Taking training on infection prevention was not found to be statistically significant on multivariable analysis in this study. But, the OR suggests that not having training may be associated with an 80% increased odds of injury in the last one year. In the presence of increased sample size, this could have been statistically significant. Similarly, this finding goes in line with previous findings [14, 16] in which training for workers seem to be not necessarily brought about protection from injury exposure. The reason for this may be: (i) knowledge gained may not necessarily be transferred into practice of preventive measures or knowledge received may not be sufficient. (ii) Those who participated on the infection prevention training may be other workers who are working as administrators rather as healthcare providers (i.e the training missed the personnel under risk of injury). (iii) The training might be given after the workers sustained the injury. (iv) The provided training may be more of theoretical than practical. Lastly, the sample size might not sufficient enough to detect the differences.

Since the study was conducted among randomly selected healthcare professionals, laundry workers and janitors or cleaners, it might be generalized to all healthcare professionals and other individuals who had direct contact with patients or equipments used on patients working in the study hospitals. But in interpreting the results of this study, taking the limitations into consideration is important. Since the study was based on self-reported data in estimating the prevalence of occupational NSSI exposure; a common threat to the validity of the self-report that can lead to information bias such as social desirability and recall bias. In addition, a cross-sectional study by its nature cannot establish temporal cause and effect relationship to identify the risk factors. The sample size used might not be large enough to detect the difference of the occurrence of the outcome variable on the explanatory variables which are declared as not statistically significant.

## Conclusions

This study revealed that more than one third of the study respondents had experienced NSSI at least once in their lifetime. Even though the past one year prevalence is lower, workers are affected by occupational NSSI at the study area. The study identified the presence of suboptimal practices that put both HCWs and patients at significant risk of contracting occupational infections. Needle recapping was the main predictor for experiencing occupational NSSI in the past one year.

Based on the findings of the study the following recommendations are forwarded to the stakeholders: Healthcare authorities in the study area should arrange training for HCWs and provision of infection prevention equipments. Health policy makers should formulate strategies to improve the working condition for healthcare workers and increase their adherence to universal precautions. Furthermore, regular reporting, follow up and evaluation of occupational injury exposures need to be introduced. Follow up study is needed to determine the actual incidence of NSSI exposure and to which type disease workers are exposed.

## Supporting Information

S1 File Questionnaire. (DOCX)

## References

[1] Deisenhammer S, Radon K, Nowak, D, Reichert J. Needlestick injuries during medical training. Journal of Hospital Infection. 2006; 63: 263–267. 16650505 10.1016/j.jhin.2006.01.019

[2] De Laune S. Risk reduction through testing, screening and infection control precautions with special emphasis on needle stick injuries. Infect Control Hosp Epidemiology. 1990; 11(10):563–565. 2230047

[3] African News letter. Occupational Health and Safety. 2010; 20:20–2. Available: http://www.ttl.fi/AfricanNewsletter.

[4] Rapiti E, Prüss ÜA, Hutin Y. Sharps injuries: assessing the burden of disease from sharps injuries to health-care workers at national and local levels. Geneva, WHO Environmental Burden of Disease Series. 2005; Issue No 11.

[5] Singhal V, Bora D, Singh S. Hepatitis B Virus in health care workers: Indian scenario. Journal of Laboratory Physicians. 2009; 1(2): 41–48. 10.4103/0974-2727.59697 21938248 PMC3167966

[6] Sagoe MC, Pearson RD, Perry J, Jagger J. Risks to health care workers in developing countries. New England J Med. 2001; 345 (7): 538–41. 11519511 10.1056/NEJM200108163450711

[7] Simonsen L, Kane A, Lloyd J, Zaffran M, Kane M. Unsafe injections in the developing world and transmission of bloodborne pathogens: a review. Bull World Health Organ. 1999; 77:789–800. 10593026 PMC2557743

[8] Nwankwo TO, Aniebue UU. Percutaneous injuries and accidental blood exposure in surgical residents: awareness and use of Prophylaxis in relation to HIV. Niger J Clin Pract. 2011; 14(1): 34–37. 10.4103/1119-3077.79237 21493989

[9] Sofola OO, Uti OG. Hepatitis B virus infection and prevention in the dental clinic: Knowledge and factors determining vaccine uptake in a Nigerian dental hospital. Nigerian Quarter Journal Hosp Med. 2008; 18(3):145–148. 19062478

[10] Stewardson DA. Occupational exposures occurring among dental assistants in a UK dental school. Primary dental care. 2003; 10:23–26. 12621857 10.1308/135576103322504076

[11] Kurt V, Donovan M, Tazhmoye C, Ruby L, Alexander L, Rachael I. Prevalence of Injuries and Reporting of Accidents among Health Care Workers at the University Hospital of the West Indies, Jamaica. International Journal of Occupational Medicine and Environmental Health. 2010; 23(2):133–143. 10.2478/v10001-010-0016-5 20630834

[12] Hanafi M, Mohamed AM, Kassem MS, Shawki M. Needle stick injuries among health care workers of University of Alexandria hospitals. Eastern Mediterranean Health Journal. 2011; 17(1):26–35. 21735798

[13] Kakizaki M, Ikeda N, Ali M, Enkhtuya B, Tsolmon M, Shibya K, et al Needle stick and sharps injuries among health care workers at public tertiary hospitals in an urban community in Mongolia. BMC Research Notes. 2011; 4:184 10.1186/1756-0500-4-184 21672224 PMC3125346

[14] Lulie W, Emebet A, Medihanit T, Hanna F, Dereje B, Muluken A. Factors associated with needle stick and sharp injuries, among healthcare workers in Felege Hiwot Referral Hospital, Bahir Dar, Northwest Ethiopia. Int J Infect Control. 2013; 9(4):1996–9783.

[15] Ali G, Abasalt B, Pegah L, Amin A. Risk Factors of Needlestick and Sharps Injuries among Healthcare Workers. International J of Hospital Research. 2013; 2(1):31–38.

[16] Reda AA, Fisseha S, Mengistie B, Vandeweerd JM. Standard Precautions: Occupational Exposure and Behavior of Health Care Workers in Ethiopia. PLOS ONE. 2010; 5(12): e14420 10.1371/journal.pone.0014420 21203449 PMC3009714

[17] Berhanu EF. Prevalence and determinant factors for Sharp Injuries among Addis Ababa Hospitals Health Professionals. Science Journal of Public Health. 2013; 1(5):189–193.

[18] Goswami M, Patel P, Nayak S, Mehta HK, Shah R, Devmurari D, et al Needle Stick and Sharp Instruments Injuries among Health Care Providers at Cardiology Institute, Ahmedabad. National J of Community Medicine. 2010; 1(2):114–117.

[19] Prisca OA, Busola TO. Exposure to work-related sharp injuries among nurses in Nigeria. Journal of Nursing Education and Practice. 2014; 4(1): 229–236.

