# Supplementary material for: Factors Associated with Occupational Needle Stick and Sharps Injuries among Hospital Healthcare Workers in Bale Zone, Southeast Ethiopia
Source: PLoS One. 2015 Oct 15;10(10):e0140382. doi: 10.1371/journal.pone.0140382 (PMC4607483; doi:10.1371/journal.pone.0140382)
Supplement: S1 File — (DOCX) [file pone.0140382.s001.docx]

**Madawalabu University, Research and Community Service Office**

Survey questionnaire on **occupational needle sticks and sharps injuries among hospital healthcare workers in Bale Zone, Oromia regional state, Southeast Ethiopia**

Questionnaire ID:------------------------- Hospital name --------------------------------

**I. Participant’s information sheet**

**Greeting**: Good morning/afternoon

My name is---------------------------------------I am working on behalf of a research team (project), in Madawalabu University. I would like to ask if you are voluntary to answer questions which take around 20 munities about **occupational needle sticks and sharps injuries among hospital healthcare workers in Bale Zone.** The participants of this study will not gain any direct benefit for being they participated. Your genuine responses that you are going to give us is very important to identify problems related to occupational injuries among hospital healthcare workers and design injury prevention and control programs for healthcare workers in hospitals and in general to our country. You are selected randomly to be participant of this study. The study has no any risk for the participants and interview will be private to make safe participants from any fear. You have full right in participating or not participating and you can also stop participating in the study at any time. You can also skip any question which you want to respond. You can ask any question which is not clear for you at any time. Any information you forwarded will be kept confidential and your name will not be specified.

**II. Informed consent**

I have read this form or it has been read to me in the language I comprehend and understand all conditions stated above. Are you willing to participate in this study?

1. No (Say Thank you) 2. Yes 🡪 continue your interview

**Name of principal investigator**: Tolesa Bekele;

**Address:** Cell phone: +251910652069; E-mail: [tolesa2003@yahoo.com](mailto:tolesa2003@yahoo.com)

**Name of institution:** Madawalabu University, College of Medicine and Health Sciences

**Address**: Bale Goba, Ethiopia

**Tel. No**: +251-22-6610559

Result of interview: 1. Completed 3. Refused. 4. Partially completed

**English version questionnaire**

**Part I: Socio-demographic characteristics of the participants**

| **No.** | **Question** | **Choices** | **Remark** |
| --- | --- | --- | --- |
| 101 | What is your working hospital? | 1. Ginir 2. Goba 3. Delo mena 4. Robe |  |
| 102 | What is your sex | 1. Male 2. Female |  |
| 103 | How old are you?(age in years) | Enter# _______________ |  |
| 104 | What is your marital status? | 1. Married 2. Single 3. Widowed 4. Divorced 5. Separated |  |
| 105 | What is your ethnicity? | 1. Oromo 2. Amhara 3. Tigrie   4. Others (**specify**)_______________ |  |
| 106 | What is your religion? | 1. Orthodox 2. Muslim 3. Protestant 4. Catholic   5. Others (**specify**)_________________ |  |
| 107 | What is your educational level? | 1. Grade (1-4th) 2. Grade (5-8^th^ ) 3. Grade (9-12th) 4. Grade 12^+^ 5. College diploma or above |  |
| 108 | What is your monthly income? | ___________Ethio.birr |  |

**Part II. Work related information**

| No | | Question | Choices | Remark |
| --- | --- | --- | --- | --- |
| 110 | | What's your job category? | 1. Nurse 2. Internist 3. Surgeon 4. Gynaecologist 5. Pedatritian 6. General practitioner (GP) 7. Midwife 8. Anaesthesia 9. Health officer 10. Clinical laboratory worker 11. Laundry worker 12. Waste handlers /cleaners   13. Other, describe______________________ |  |
| 111 | | What is your current working section/department? | 1. Emergency unit 2. Paediatric ward 3. Maternity Ward 4. Operation room (OR) 5. Medical ward 6. Surgical ward 7. Waste handlers unit 8. Laboratory unit 9. OPD 10. Laundry room 11. Others; Specify _________________ |  |
| 112 | | For how long have you been in this working section? | ________ Years |  |
| 113 | | What is your total services year in health facility? | ___________ Years |  |
| 114 | | Do you work in shifts? | 1. Yes 2. No |  |
| 115 | | How many hours do you work in the hospital per day? | 1. 8 hours 2. 12 hours 3. 24 Hours 4. Other specify __________________ |  |
| **Part III. Participants’ Knowledge about needle stick and sharps injury**   \| No. \| Questions \| Choices \| Remark \| \| --- \| --- \| --- \| --- \| \| 116 \| \| Do you know about the risk of needle stick/sharp injury? \| \| --- \| \| 1. Yes 2. No \| Go to  Q118 \| \| 117 \| \| How do you rate the risk of needle stick/ sharps injuries \| \| --- \| \| 1. Not risky 2. Low risk 3. Moderate risk 4. High risk 5. I do not know \|  \| \| 118 \| \| Do you think needle stick or sharps injury is avoidable? \| \| --- \| \| 1. Yes 2. No \| Go to  Q120 \| \| 119 \| If “**Yes**” how? \|  \|  \| \| 120 \| \| Do you think that disease can be transmitted by needle or sharp injuries? \| \| --- \| \| 1. Yes 2. No \| Go to  Q122 \| \| 121 \| Which of the following diseases can be transmitted through blood and body fluids b/c of needle or sharps injury? \| 1. Human immunodeficiency virus (HIV) 2. Hepatitis B virus (HBV) 3. Hepatitis C virus (HCV) 4. Malaria \|  \| \| 122 \| Do you recap needles after use? \| 1. Yes 2. No \| Go to  Q124 \| \| 123 \| How do you recap the needles after use? \| 1. With one hand 2. With two hands \|  \| \| 124 \| Which of the following factors do you think are contributing to needle stick/sharp injury in your hospital? \| 1. Lack of personal protection equipment 2. Inadequate hand washing facility 3. Excess work load 4. Over crowded work place 5. Other(specify)________________ \|  \|   **Part IV**: - **In reference to your most recent needle stick and/or sharp objects injury, please answer the following questions.** | | | | |
| 125 | Have you ever experienced/ faced needle stick and/or sharps injuries at your work place? | | 1. Yes 2. No | Go to  Q 142 |
| 126 | How many times have you been injured by needles stick or sharp objects? | | 1. Once 2. twice 3. 3 times and above |  |
| 127 | If **Q 125** is “**Yes**” when was the time you have experienced the recent injury? | | 1. In the past 12 months (**this year**) 2. In the past 24 months(**last year**) 3. In the past 3 years and before |  |
| 128 | What were the **reasons** for your needle stick or sharps injury?  (**circle all that apply**) | | 1. Opening the needle cap 2. Drawing blood from patient 3. During injection on patients 4. Recapping 5. Washing the instrument 6. Disassembling needle and syringe after use 7. Attempting to bend the needle 8. Needle penetrating cap 9. Accidental uncapping of needle already recapped 10. Transferring blood into test tube 11. By needle after use, before disposal 12. Injured by improperly disposed needle 13. While introducing the needle into a disposal box 14. Injured by needle protruding from disposal box 15. Injured accidentally by carelessness held by colleague 16. Sudden movement of the patient 17. Lack of concentration 18. During gown collection for laundry 19. Lack of experience 20. Inadequate disposal equipment 21. During handling and collection of wastes 22. Cleaning of “infectious waste” 23. Cleaning of “non-infectious waste” 24. Other Specify ________________ |  |
| 129 | Where did the injury occur? | | 1. Emergency unit 2. Paediatric ward 3. Maternity Ward 4. Operation Theatre Unit 5. Medical ward 6. Surgical ward 7. Waste handlers unit 8. Laboratory unit 9. OPD 10. Others(Specify) _________________ |  |
| 130 | What type of item caused this injury? (including needles and  sharps, **circle all that apply**) | | 1. Syringe needle 2. Angiocath needle 3. Butterfly needle 4. Intravenous catheter 5. Air-induction needle 6. Ampoule 7. Tapping needle 8. Blood glucose lancet 9. Phlebotomy needle 10. Suture needle 11. Scalpel blade 12. Glass item 13. Other (specify)______________________ |  |
| 131 | How deep was your needle stick/sharp injury? | | 1. Deep 2. Superficial |  |
| 132 | Does the needle and/or sharp which injured you been used on any patient? | | 1. Yes 2. No | Go to  Q135 |
| 133 | Do you know the disease **type** of the patient from which the needle stick injury occurred? | | 1. Yes 2. No | Go to  Q135 |
| 134 | If “**Yes**” to **Q 133**, does the patient have of the following infectious diseases? | | 1. Patient had hepatitis B virus 2. Patient had hepatitis C virus 3. Patient had both hepatitis B & C virus 4. Patient had syphilis 5. Patient had HIV 6. Patient had other disease other than the above |  |
| 135 | Have you taken any action by yourself after the injury? | | 1. Yes 2. No | Go to  Q139 |
| 136 | What did you do immediately after the injury?  (***Circle that all apply***) | | 1. Washing with soap and water 2. Wash with iodine or alcohol solution 3. Get tested for HIV 4. Take post exposure prophylaxis (PEP) 5. Take tetanus anti-toxoid (TAT) 6. Squeezing to extract more blood 7. Applying pressure to stop bleeding 8. I did not take any action 9. Other, specify____________________ |  |
| 137 | Have you ever reported the injury to infection prevention and control department? | | 1. Yes 2. No | Go to  Q139 |
| 138 | If “**Yes**” to **Q137**, When did you report the injury? | | 1. Immediately after the injury 2. Late before going off duty 3. The following days 4. More than two days after injury 5. Other specify_______________________ |  |
| 139 | If “**No”** to **Q 137** (NOT reported), **why**?  (***circle all that apply***): | | 1. I was too busy at that time 2. Needles that injured me was never used in a patient 3. sharp object was used on patient did not have infectious diseases of concern 4. My colleagues suggested that I should be all right and did not have to worry 5. I did not know I needed to report the event 6. I did not know how to report 7. I did the report when I was injured in the past 8. I had either hepatitis B antigen or antibody 9. I had been injured for too many times 10. Other (specify)______________ |  |
| 140 | Did you receive medical care after injury? | | 1. Yes 2. No | Go to  Q142 |
| 141 | If “**Yes**” to **Q140**, what was done for you? | | 1. the necessary examinations were done 2. pre-test counselling was given to me 3. Post-test counselling was given to me 4. prophylactic was given and follow-up started 5. No further evaluation was needed 6. they did not give me any advice or follow-up 7. I am still waiting for further measures from the administrators 8. Other, please describe______________ |  |
| 142 | Have you ever been diagnosed for the following infectious disease? | | 1. I have not been diagnosed for any disease 2. I have been diagnosis hepatitis B 3. I have been diagnosis for hepatitis C 4. I have been diagnosis HIV |  |
| 143 | Do you disassemble used needles or sharps with your hands? | | 1. Yes 2. No |  |
| 144 | Was there protocol for reporting the needle sticks/ sharp injury in your hospital? | | 1. Yes 2. No | Go to  Q146 |
| 145 | If **“No”** why? | | ______________________________ |  |
| 146 | Were safety **guidelines** available at your working environment? | | 1. Yes 2. No | Go to  Q148 |
| 147 | If **“No”** why? | | ___________________________ |  |
| 148 | Was safety box available at your work place? | | 1. Yes 2. No | Go to  Q150 |
| 149 | If **“No”** why? | | _______________________________ |  |
| 150 | Do you regularly apply universal precautions? | | 1. Yes 2. No |  |
| 151 | Do you regularly use personal protective equipments? | | 1. Yes 2. No | Go to  Q153 |
| 152 | If “**Yes”** to **Q 152**, How often do you use? | | 1. Always 2. Sometimes 3. Occasionally |  |
| 153 | Have you received any form of training on **infection prevention**? | | 1. Yes 2. No |  |
| 154 | Have you ever vaccinated against Hepatitis B virus? | | 1. Yes 2. No |  |

**THANK YOU !**
